# Supplementary material for: Causal Relationship between Aging and Anorexia Nervosa: A White-Matter-Microstructure-Mediated Mendelian Randomization Analysis
Source: Biomedicines. 2024 Aug 16;12(8):1874. doi: 10.3390/biomedicines12081874 (PMC11351342; doi:10.3390/biomedicines12081874)
Supplement: Supplementary file 1 [file biomedicines-12-01874-s001.zip › Supplementary Figure/Figure S8/Leaveoneout plot of MR analysis for TL to ubm-b-1923.pdf]

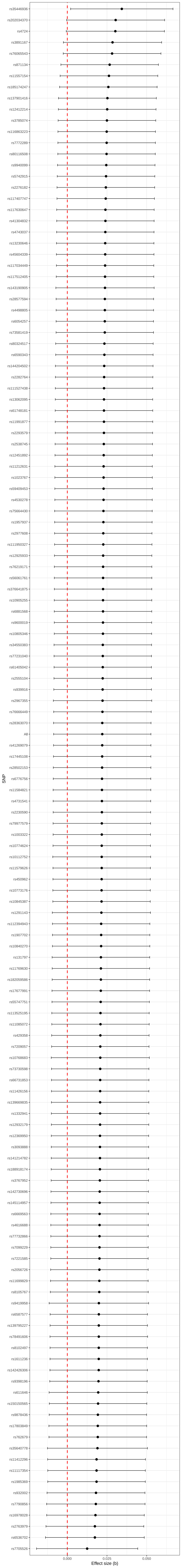

rs35446938
rs202034370
rs4724
rs3891167
rs76065543
rs871134
rs11557154
rs185174247
rs137901416
rs12412214
rs3785074
rs116863223
rs7772289
rs80116508
rs9940099
rs5742915
rs2276182
rs117407747
rs117630647
rs41304832
rs4743037
rs13230646
rs45604339
rs117034449
rs117512405
rs143190905
rs28577594
rs4498805
rs6054257
rs73581419
rs80324517
rs6590343
rs144204502
rs2292764
rs111527438
rs13062095
rs61748181
rs11991877
rs2293579
rs2538745
rs12451892
rs11212631
rs1023767
rs59409453
rs4530278
rs75664430
rs1957937
rs2977608
rs111950327
rs12925933
rs76219171
rs56061761
rs376641875
rs10905255
rs6881568
rs9600019
rs10805346
rs34550383
rs77231040
rs61405042
rs2555104
rs939916
rs2967355
rs76666449
rs28363070
All
rs41269079
rs17445108
rs28502153
rs6776756
rs11584821
rs4731541
rs2230590
rs79977579
rs1003322
rs10774624
rs10112752
rs15579626
rs450962
rs10773176
rs10845387
rs1291143
rs112394943
rs1907702
rs10840270
rs131797
rs11769630
rs182059586
rs17677991
rs55747751
rs113525195
rs11085072
rs429358
rs7209057
rs10768683
rs73730598
rs66731853
rs11428156
rs139669835
rs1332941
rs12932179
rs12369950
rs3093888
rs141214782
rs188918174
rs3767952
rs142730696
rs145114957
rs6669563
rs4616688
rs77732866
rs7099229
rs7221585
rs2056726
rs11699829
rs8105767
rs9419958
rs6587577
rs139795227
rs78491606
rs8102497
rs1611236
rs142426306
rs9398196
rs611646
rs150150565
rs9878436
rs17803849
rs762679
rs35640778
rs11412296
rs11117354
rs1985369
rs932002
rs7790858
rs16978028
rs2763979
rs6536702
rs7705526
SNP
0.000
0.025
0.050
Effect size (b)
